# Early Detection of Postharvest Potato Tuber Dry Rot Based on Hyperspectral Imaging and a Dual-Branch ResNet12-SE Spatial–Spectral Fusion Network

**DOI:** 10.3390/jof12090702

**Published:** 2026-09-20

**Authors:** Hanwen Cao, Jiahui Liu, Tao Liu, Mingmin Zhao, Huali Xue, Min Hao

**Affiliations:** 1 College of Mechanical and Electrical Engineering, Inner Mongolia Agricultural University, Hohhot 010018, China; 2 Key Laboratory of the Development and Resource Utilization of Biological Pesticide in Inner Mongolia, Inner Mongolia Agricultural University, Hohhot 010018, China; 3 College of Science, Gansu Agricultural University, Lanzhou 730070, China

**Keywords:** *Fusarium sulphureum*, latent internal decay, crop quality, reflectance signatures, attention mechanism, recurrent spectral modeling, quality screening

## Abstract

Potato tuber dry rot is a major postharvest decay caused predominantly by *Fusarium* spp. During early infection, external symptoms may be absent even when faint internal browning, localized dehydration, and tissue structure changes have begun. Manual inspection, destructive cutting, and culture- or molecular-based assays are therefore poorly suited to rapid, nondestructive, high-throughput screening. We developed a near-infrared hyperspectral imaging method that combines spatial and spectral representations in a dual-branch ResNet12-SE network. The working dataset contained 1725 labeled records (862 healthy and 863 early-infected records); records were assigned to subsets by tuber identifier, and an infected volume ratio below 5% was used as an operational early infection threshold. The calibrated model input was a 224-band, 224 × 224 hyperspectral cube. The spatial branch used a ResNet-12 backbone with spatial squeeze-and-excitation (SE), whereas the spectral branch used spectral SE followed by bidirectional long short-term memory (BiLSTM). The two feature vectors were concatenated for binary classification. In the single-split test, the proposed model achieved 98.22% accuracy, compared with 90.67% for the ResNet-12 baseline and 97.43% for Vision Transformer. Preprocessing, principal component image, local binary pattern, and gray-level co-occurrence matrix analyses provide complementary interpretation of the spectral and spatial responses. The results support the feasibility of hyperspectral screening under the controlled laboratory protocol and define the validation work required before broader deployment.

## 1. Introduction

Potato (*Solanum tuberosum* L.) is an important staple and industrial crop whose postharvest quality affects food security, processing value, and farm income [1,2,3]. High moisture content and susceptibility to wounding make tubers vulnerable to infection during storage. Dry rot caused by *Fusarium sulphureum* is among the most important postharvest diseases reported for stored tubers in Inner Mongolia, China [2,4,5]. Infection commonly enters through wounds or natural openings and may remain subepidermal or internal before browning, dehydration, shrinkage, cavity formation, and decay become visible. *F. sulphureum* can also produce trichothecenes, which increases the importance of early quality screening [5]. A rapid, nondestructive method that detects latent tissue changes would therefore support disease management and loss reduction during storage and sorting.

Current dry rot detection relies on visual inspection, destructive cutting, culture-based identification, or molecular assays. Visual grading is subjective and difficult to scale, whereas laboratory assays require time, specialist handling, and destructive sampling. Hyperspectral imaging combines continuous reflectance spectra with spatial information and has been used to distinguish agricultural materials such as ginkgo fruit varieties [6]. It can reveal changes in moisture, pigments, cellular structure, and chemical composition that accompany disease. Previous studies have used deep learning for potato leaf diseases [7,8], spectral preprocessing and wavelength selection for potato disease classification [9], and hyperspectral or multimodal imaging for soybean, cotton, melon, strawberry, viral, and other plant diseases [10,11,12,13,14,15]. Hyperspectral sensing has also been applied to quality assessment of agricultural and medicinal materials [16]. These reports motivate its use for internal quality evaluation of stored tubers.

Spatial texture and structural features provide a second description of disease response. Convolutional and hybrid models have been used with RGB, multispectral, and hyperspectral images for crop disease recognition [17,18,19,20,21,22]. Such features can describe lesion morphology, local gray-level transitions, and tissue heterogeneity. In early potato dry rot, however, the affected region may be small, and external contrast may be weak. Visible images or handcrafted texture descriptors alone may therefore miss part of the disease signal, whereas the spectral dimension can provide complementary information about internal tissue changes.

Hyperspectral data contain correlated spectral bands and spatial structure, so a fusion model should preserve both forms of information while accounting for their different organizations. Full-spectrum hyperspectral CNNs have detected physiological disorders [23], and multimodal fluorescence or reflectance systems have improved early disease screening [24]. Spatial–spectral and plant trait fusion has also been explored in UAV imagery [25], while generative models have been used to strengthen subtle spectral and spatial features [26]. Most of these studies focus on leaves, canopies, or diseases with visible symptoms. Early postharvest tuber dry rot remains less studied, and simple feature concatenation does not explicitly model dependencies along the spectral axis. A dual-branch design with attention and recurrent spectral modeling is therefore motivated by the structure of the data.

This study aimed to develop and evaluate a nondestructive early detection method for postharvest potato dry rot using near-infrared hyperspectral imaging. The objectives were to (1) characterize spectral responses and spatial texture changes associated with early infection; (2) construct and evaluate a dual-branch ResNet12-SE spatial–spectral fusion model; (3) quantify the observed contributions of spatial SE, spectral SE, and BiLSTM through ablation experiments; and (4) compare the proposed model with ResNet variants, EfficientNetV2-S, and Vision Transformer under a common input representation. The 5% infected volume ratio was used as an operational study threshold for the binary task, and its pathological generality is addressed as a validation question rather than assumed a priori.

## 2. Materials and Methods

### 2.1. Experimental Materials and Sample Preparation

Potato tubers of the cultivar “Favorite” were purchased from a local agricultural market in Hohhot, Inner Mongolia, China. Fresh tubers from one purchase batch were selected to reduce variation in maturity and storage history. Tubers were similar in size and shape and had no visible lesions or obvious mechanical damage. They were washed and immersed for 5 min in 0.5% sodium hypochlorite solution (Tianjin Xinbote Chemical Co., Ltd., Tianjin, China). The tubers were then rinsed three times with deionized water, air-dried, and assigned unique identifiers.

*Fusarium sulphureum* was provided by Gansu Agricultural University and cultured on potato dextrose agar (PDA) for 5–7 days. The PDA medium was prepared in the laboratory using anhydrous glucose and agar. Anhydrous glucose was supplied by Tianjin Aopusheng Chemical Co., Ltd. (Tianjin, China), and agar by LABLEAD (Beijing, China). Sterile water was supplied by Beijing Solarbio Science & Technology Co., Ltd. (Beijing, China). Tween 80 was supplied by Tianjin Fengchuan Chemical Reagent Technology Co., Ltd. (Tianjin, China). Sterile water containing 0.05% Tween 80 was added to the culture, and mycelia and spores were gently scraped to prepare a suspension. Spore concentration was measured with a hemocytometer and diluted to 1 × 10^6^ spores/mL.

For inoculation, two symmetrical equatorial points were selected on each tuber; sterile needles were used to make holes of approximately 3 mm depth and 3 mm diameter, and 20 μL of suspension was injected into each hole. Tubers were incubated at 25 ± 2 °C and 90% relative humidity, with hyperspectral images collected every 48 h during early development. Healthy controls were defined operationally as records with no detectable lesion after post-acquisition sectioning. These records were classified as lesion-negative controls; their puncture and inoculation status was not separately documented. A dedicated sterile mock inoculation/mechanical wound control is required in future experiments to separate handling effects from pathogen-associated responses.

### 2.2. Hyperspectral Data Acquisition and Label Confirmation

**Figure 1:**
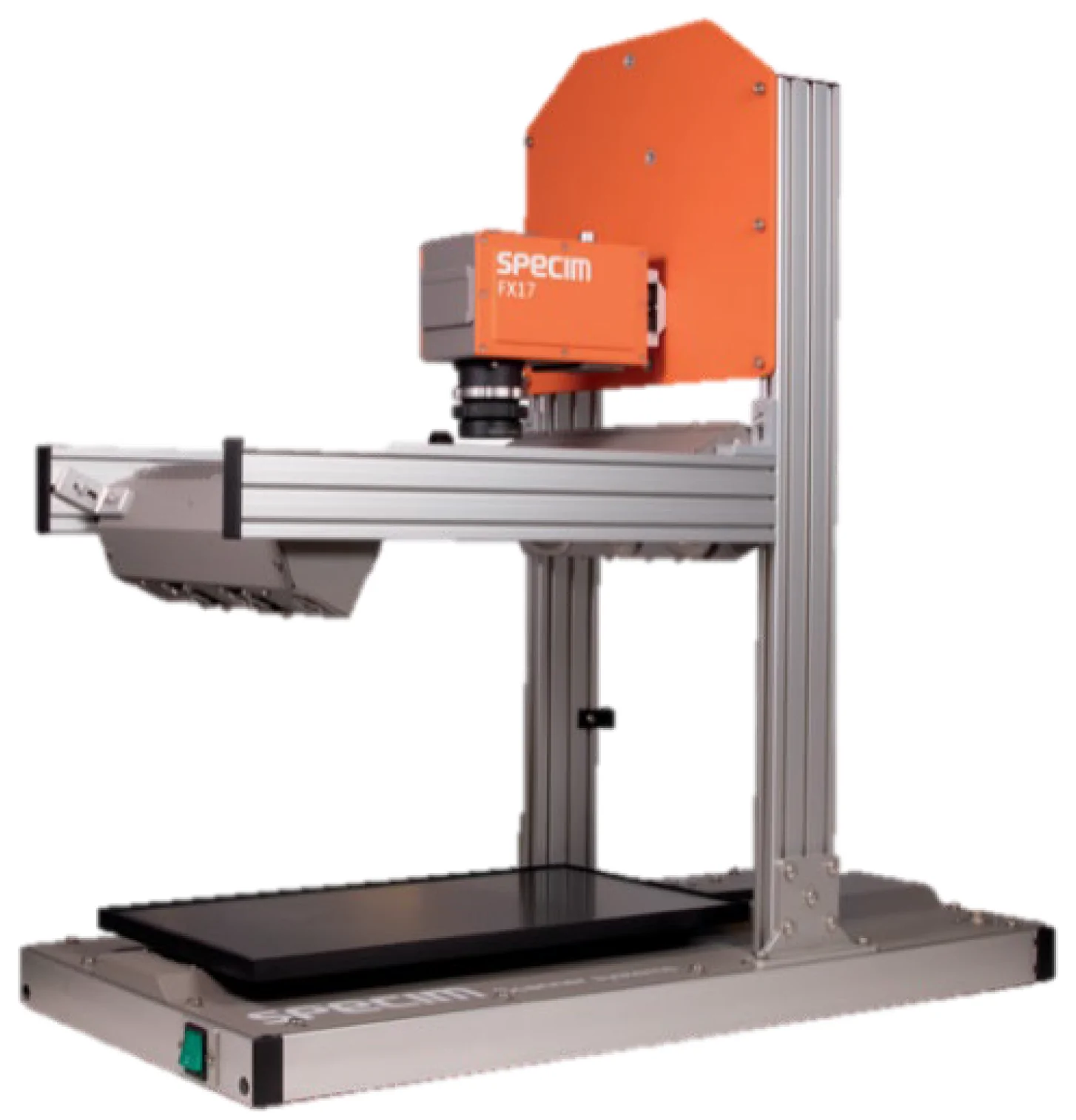
Specim FX17 hyperspectral camera and scanning platform used for tuber image acquisition. The orange camera housing is mounted above the scanning stage.

A Specim FX17 near-infrared hyperspectral imaging system (Specim, Spectral Imaging Ltd., Oulu, Finland) acquired the tuber images (Figure 1). The system comprised a camera, a translation stage, and two halogen lamps and was controlled using LUMO Scanner V1.2 software (Specim, Spectral Imaging Ltd., Oulu, Finland). Images were acquired over the nominal 900–1700 nm range with an approximately 3.5 nm sampling interval. The calibrated cube used by the deep learning models contained 224 spectral channels and was resized to 224 × 224 pixels. The system was warmed for 30 min; lamps illuminated the stage symmetrically at 45°, the stage speed was 5 mm/s, and the exposure time was 25 ms.

Before each acquisition, white and dark reference images were recorded to correct sensor dark current and illumination nonuniformity. The calibrated reflectance at wavelength *λ* was calculated with Equation (1).

(1) $$\rho_{\lambda }=\frac{I_{\lambda }-D_{\lambda }}{W_{\lambda }-D_{\lambda }}$$

Here, *I_λ_* is the raw hyperspectral intensity at wavelength *λ*, *D_λ_* is the dark reference intensity, *W_λ_* is the white reference intensity, and *ρ_λ_* is the calibrated reflectance. All subsequent analyses used the calibrated reflectance data. Regions of interest (ROIs) and their mean spectra for the conventional spectral analysis were extracted using ENVI 5.6.

**Figure 2:**
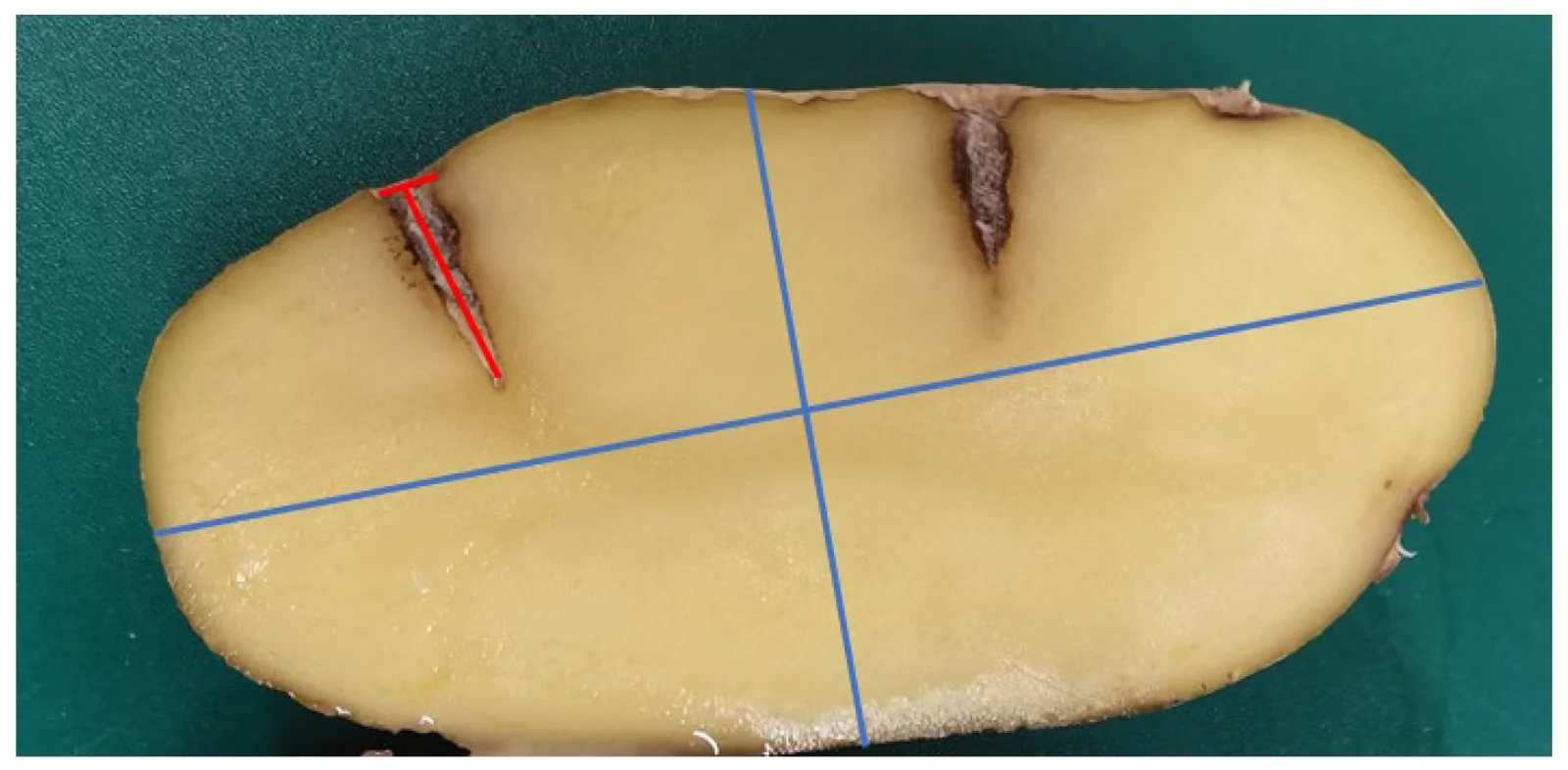
Measurement of tuber and lesion dimensions on a cut surface. Blue lines mark the principal tuber dimensions visible in the section, and red lines mark the lesion radius and depth. Tuber length, width, and thickness were used for the ellipsoidal volume estimate. Effective lesion depth was calculated by subtracting the approximately 3 mm inoculation hole depth from the measured lesion depth.

After acquisition, each tuber was cut through the inoculation site. The maximum transverse lesion radius (*r*_l_) and maximum longitudinal lesion depth (*h*_l_) were measured on the cut surface with a ruler or vernier caliper (Figure 2). The approximately 3 mm inoculation hole (*h*_0_) was treated as procedure-related damage and subtracted from the measured depth; the effective pathogen-associated depth was calculated with Equation (2).

(2) $$h_{eff}=h_{l}-h_{0}$$

Here, *h*_eff_ is the effective pathogen-associated lesion depth and *h*_0_ = 3 mm is the initial depth of the artificial inoculation hole.

The lesion was represented by an equivalent right circular cone with base radius *r*_l_ and effective depth *h*_eff_. Its estimated volume was calculated with Equation (3). The approximation treats the measured maximum transverse radius as an equivalent circular base and does not imply that the biological lesion is conical.

(3) $$V_{lesion}=\left(\frac{1}{3}\right)\;\pi \;{r_{l}}^{2}h_{eff}$$

Here, *V*_lesion_ is the estimated lesion volume. The approximation uses the measured maximum transverse radius as an equivalent circular base and uses the inoculation hole correction only in *h*_eff_.

The whole tuber was represented by an equivalent ellipsoid using its maximum length (*L*), width (*W*), and thickness (*T*), as shown in Equation (4).

(4) $$V_{tuber}=(\frac{\pi }{6})\;L\;W\;T$$

The infected volume ratio was calculated with Equation (5).

(5) $$Infected\;volume\;ratio=\frac{V_{lesion}}{V_{tuber}}\times 100\%$$

The 5% cutoff was selected as an operational definition of a low-volume lesion that is likely to be externally inconspicuous in this proof-of-concept task. It is not treated as a validated pathological boundary. Continuous lesion volume distributions and threshold sensitivity analyses should be evaluated in an independent validation cohort before the cutoff is used for another cultivar, storage condition, or commercial decision.

### 2.3. Dataset Construction

The dataset contained 1725 labeled records, including 862 healthy and 863 early-infected records. The reported count is a record count; the tuber identifier was the grouping unit used for partitioning. Records, spectra, images, and derived features associated with one identifier were assigned to only one subset, so regions of interest, patches, or augmented versions from the same tuber could not cross the train, validation, and test sets. The split ratio was 7:1:2. Validation data were used for model selection, and the held-out test subset was used only for final evaluation.

### 2.4. Image Feature Extraction and Spatial-Branch Input

Principal component analysis (PCA) was used only for exploratory visualization and handcrafted texture interpretation. The SG-MSC-corrected 224-band cube, rather than three PCA channels, was supplied to the deep learning models. The first three PC images were retained to display dominant spatial variation and to calculate local binary pattern (LBP) and gray-level co-occurrence matrix (GLCM) descriptors. The conventional model results are single-split benchmarks; independent validation should estimate data-dependent transformations using only the training portion and apply them unchanged to validation and test data.

LBP and GLCM descriptors were extracted from the first three PC images to interpret local texture and gray-level co-occurrence structure. LBP captures local-neighborhood patterns, whereas GLCM summarizes contrast, correlation, energy, homogeneity, dissimilarity, and angular second moment. These descriptors were used for interpretation and conventional model benchmarks, not as substitutes for the full-band deep learning input.

### 2.5. Dual-Branch ResNet12-SE Spatial–Spectral Fusion Model

**Figure 3:**
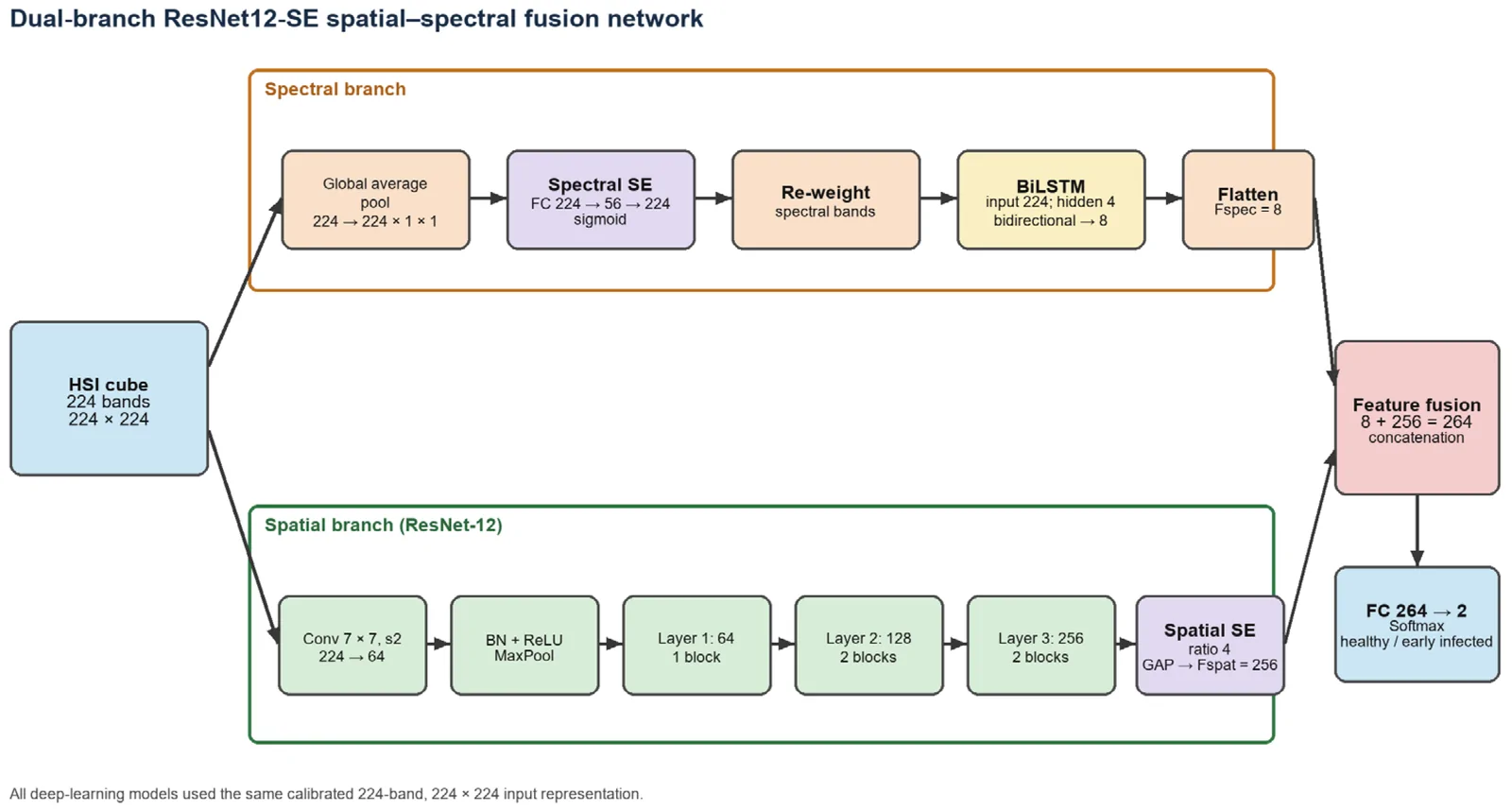
Architecture of the proposed dual-branch ResNet12-SE network. The input is a calibrated 224-band, 224 × 224 hyperspectral cube. The orange and green outlines identify the spectral and spatial branches, respectively; purple boxes denote SE modules, the yellow box denotes BiLSTM, and the pink box denotes feature fusion. The spatial branch produces *F*_spat_ = 256 and the spectral branch produces *F*_spec_ = 8, yielding 264 features before binary classification. HSI, hyperspectral imaging; SE, squeeze-and-excitation; BiLSTM, bidirectional long short-term memory; FC, fully connected; GAP, global average pooling; BN, batch normalization; ReLU, rectified linear unit.

The proposed dual-branch ResNet12-SE model receives the calibrated 224-band, 224 × 224 hyperspectral cube (Figure 3). Its spatial branch begins with a 7 × 7, stride-2 convolution (224 → 64), followed by batch normalization, ReLU, max pooling, and a ResNet-12 stack with channel widths 64, 128, and 256 and block counts 1, 2, and 2. A channel-wise spatial SE module with reduction ratio 4 is applied before global average pooling, producing a 256-dimensional spatial vector. The spectral branch applies global average pooling to the 224-band cube, a 224-dimensional fully connected projection, spectral SE with reduction ratio 4, and a one-layer bidirectional LSTM with input size 224 and hidden size 4 in each direction. The bidirectional output is 8-dimensional. Concatenation therefore gives 264 features, which are mapped by a fully connected layer to the two classes. ResNet-12/18/34/50, EfficientNetV2-S, and ViT comparators were also configured for the same 224-band, 224 × 224 input and the same calibrated preprocessing so that architecture and input information were not confounded.

### 2.6. Model Training and Implementation

Conventional spectral classifiers were implemented in Python 3.8 using scikit-learn 1.1.1. NumPy 1.22.3 and SciPy 1.8.0 were used for numerical and scientific computing, respectively. The deep learning models were implemented using PyTorch 2.1, with OpenCV 4.10 for image processing. Training used stochastic gradient descent (SGD) with momentum of 0.9, an initial learning rate of 0.01, cross-entropy loss, batch size 24, four data loader workers, and 150 epochs. The scheduler was StepLR with step size 10,000 and *γ* = 0.8; therefore, no learning rate step occurred within 150 epochs. The random seed was 971104. Training augmentation consisted of random flipping and random rotation; noise and random cut augmentation were disabled. Validation and test data were evaluated without augmentation. Checkpoints were saved during the final 30 epochs when validation accuracy exceeded the current best value, and the best validation checkpoint was selected. No patience-based early stopping rule was used.

For the conventional spectral benchmarks, selected bands were fixed before the final single-split comparison. For a leakage-controlled reproducible rerun, every data-dependent transformation, including scaling, PCA, or wavelength selection, should be fitted on the training portion and then applied unchanged to validation and test data. The reported conventional and deep learning results are single-split benchmarks.

### 2.7. Evaluation Metrics

The binary metrics were defined from true positives (TP), true negatives (TN), false positives (FP), and false negatives (FN), with early-infected records as the positive class. Accuracy, precision, recall (sensitivity), specificity, *F*_1_-score, and area under the receiver operating characteristic curve (AUC) were considered when the corresponding prediction scores were available. The formulas are given in Equations (6)–(10).

(6) $$Precision=\frac{TP}{TP+FP}$$

(7) $$Recall=\frac{TP}{TP+FN}$$

(8) $$Specificity=\frac{TN}{TN+FP}$$

(9) $$F_{1}=\frac{2TP}{2TP+FP+FN}$$

(10) $$Accuracy=\frac{TP+TN}{TP+TN+FP+FN}$$

### 2.8. Statistical Analysis

Descriptive statistics were used for spectral and image-derived features. For the single-split machine learning and deep learning benchmarks, accuracy was reported for every model; precision, recall, specificity, *F*_1_-score, and AUC were calculated when the corresponding class predictions or scores were available. The deep learning values represent one training run with a fixed random seed (971104), and no repeated run confidence interval or formal paired significance test was calculated. Accordingly, differences between models, including the 0.79 percentage point difference between ResNet12-SE and ViT, are interpreted as descriptive. Future validation should use group-wise repeated resampling or cross-validation at the tuber level and report confidence intervals with paired model comparisons.

## 3. Results

### 3.1. Comparison of Spectral Preprocessing Methods

**Figure 4:**
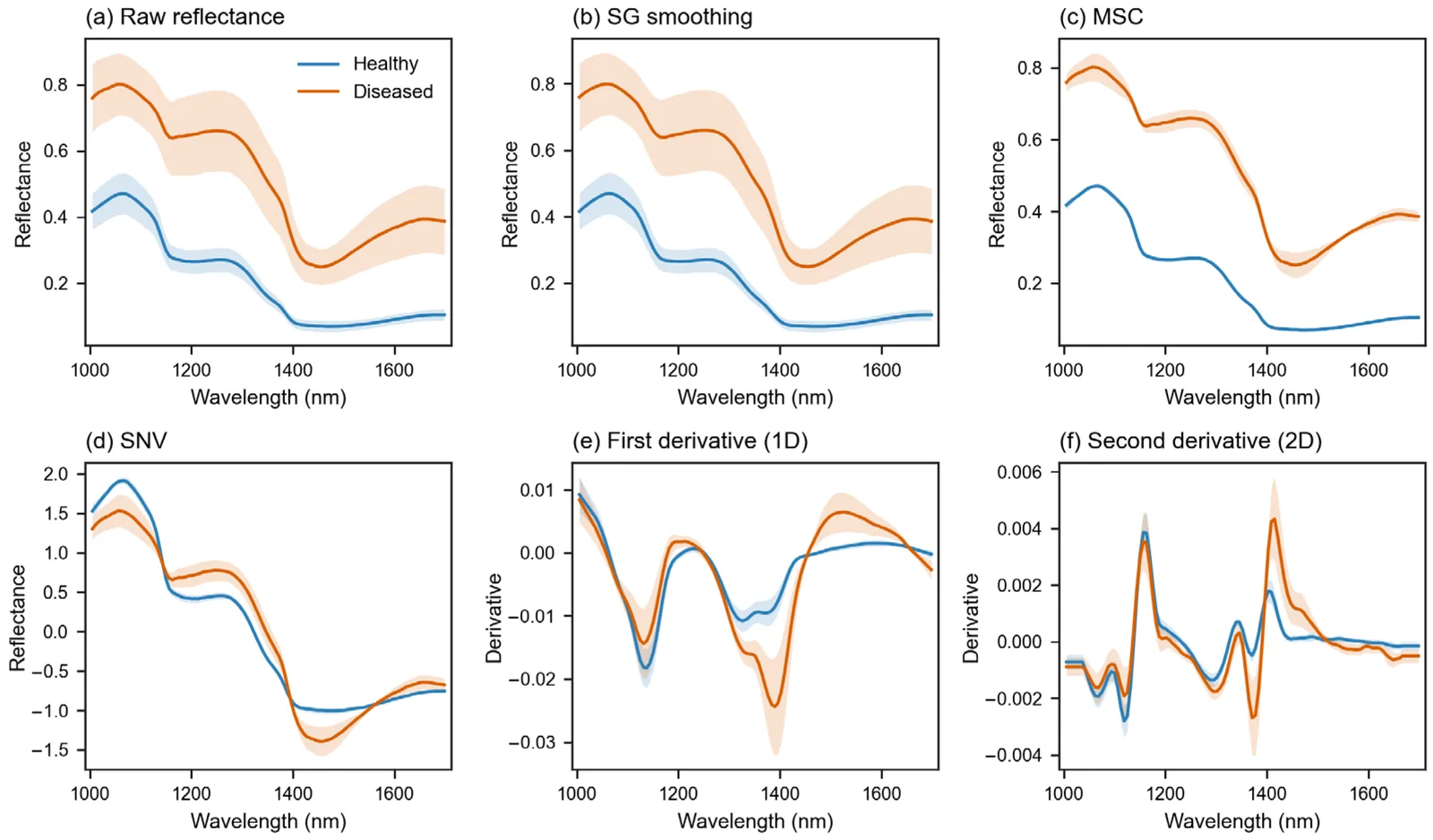
Mean spectra of healthy and early-infected records: (**a**) raw reflectance; (**b**) SG smoothing; (**c**) MSC; (**d**) SNV; (**e**) first derivative (1D); and (**f**) second derivative (2D). Blue and orange curves represent healthy and early-infected records, respectively; shaded bands represent the standard deviation across records. SG, Savitzky–Golay smoothing; MSC, multiplicative scatter correction; SNV, standard normal variate.

Mean reflectance spectra were compared for healthy and early-infected records. Figure 4 shows differences across the usable 935–1700 nm interval, including the region around the 1450 nm water absorption feature. Higher reflectance in early-infected records may reflect local dehydration, tissue disruption, browning, and altered internal scattering. These interpretations describe spectral associations and do not by themselves identify a single biochemical mechanism.

**Figure 5:**
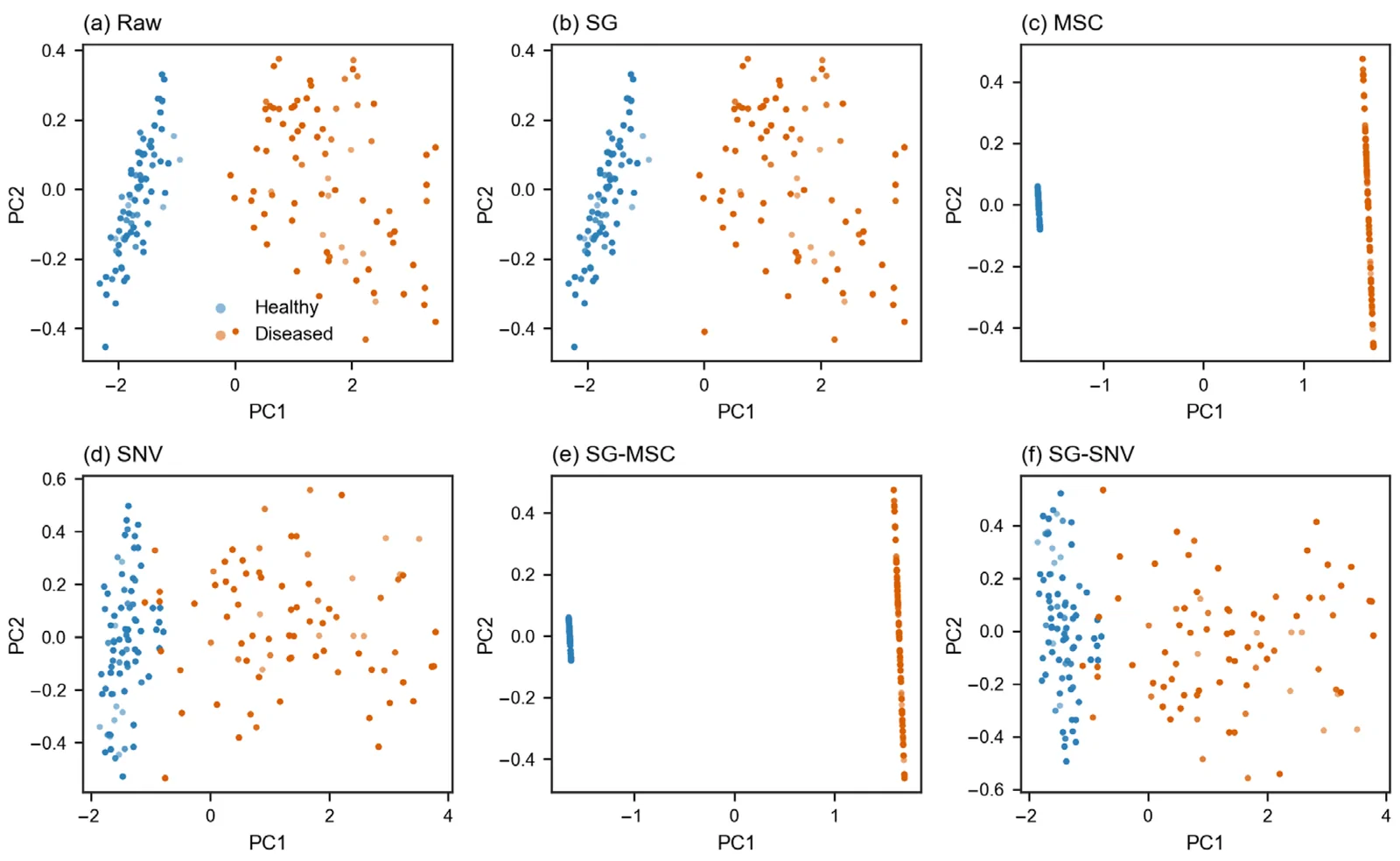
Two-dimensional PCA projections of healthy and early-infected records: (**a**) raw spectra; (**b**) SG; (**c**) MSC; (**d**) SNV; (**e**) SG-MSC; and (**f**) SG-SNV. Blue and orange points represent healthy and early-infected records, respectively. PC1 and PC2 denote the first and second principal components. PCA, principal component analysis; SG, Savitzky–Golay smoothing; MSC, multiplicative scatter correction; SNV, standard normal variate.

Noise, baseline drift, and scattering were addressed with SG, MSC, SNV, first derivative, second derivative, and their combinations. SG reduced high-frequency noise while preserving absorption profiles [27]; MSC corrected multiplicative and additive scattering effects [28]; derivative processing emphasized curvature but could amplify noise. The two-dimensional projections in Figure 5 showed a clearer separation trend after SG-MSC.

A full-spectrum support vector machine (SVM) benchmark was used to compare preprocessing choices (Table 1). SG reached 93% accuracy among single operations, MSC reached 91%, and SNV alone reached 58%. SG-MSC reached the highest observed value, 97%, in this single benchmark. The sequence was selected for later analyses because smoothing before scatter correction reduced high-frequency noise before baseline adjustment. The result is descriptive; no repeated run significance test was performed.

SG-MSC was therefore used as the common preprocessing representation for the conventional spectral analyses and for the calibrated deep learning input. Spectral features capture changes in moisture, tissue structure, and chemical composition, whereas spatial descriptors capture where texture and gray-level changes occur. The two views were retained for complementary analysis.

### 3.2. Exploratory PC Image and Texture Analysis

**Figure 6:**
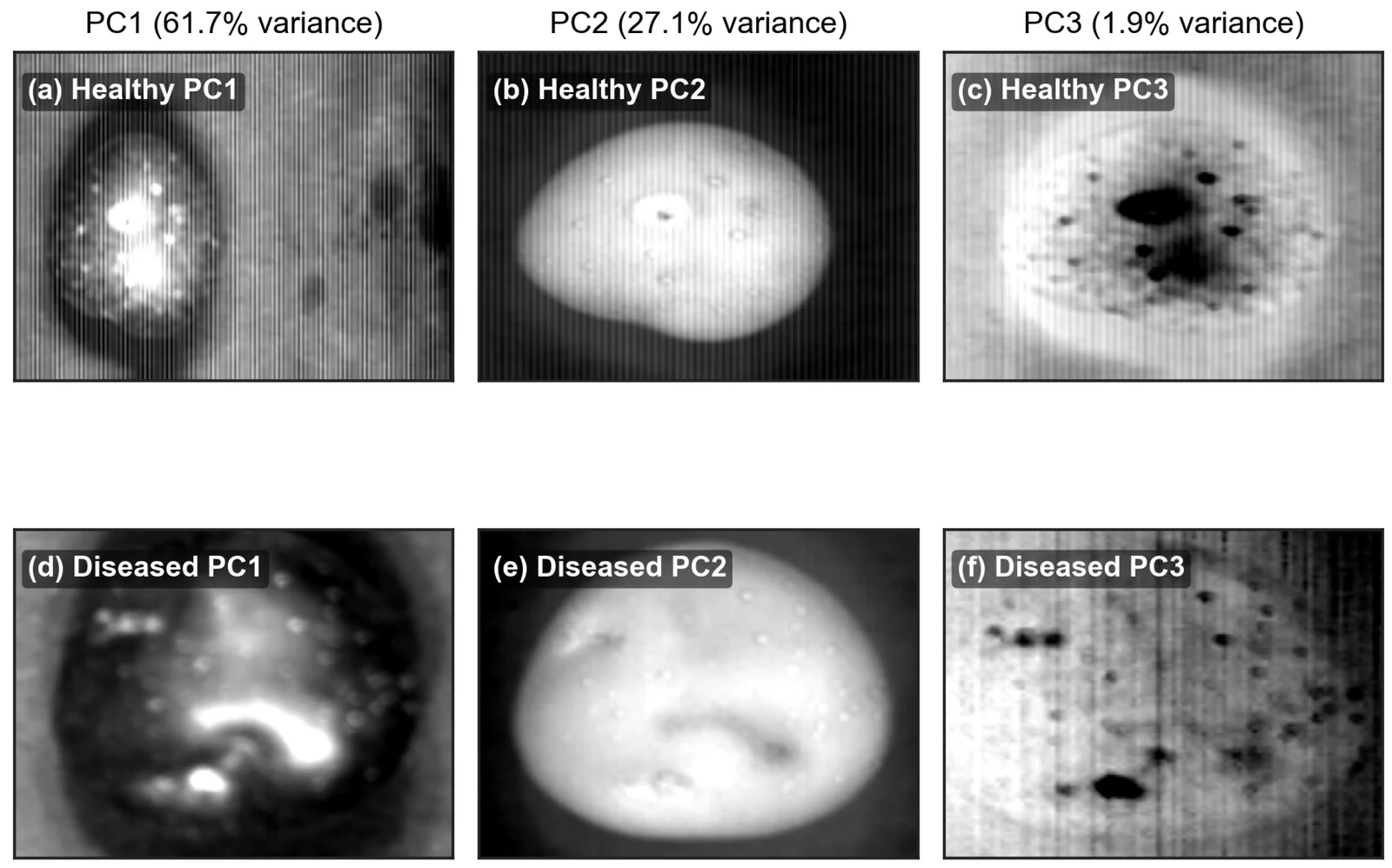
Representative principal component images: (**a**–**c**) PC1, PC2, and PC3 of a healthy tuber, respectively; (**d**–**f**) PC1, PC2, and PC3 of an early-infected tuber, respectively. The first three components explain 61.7%, 27.1%, and 1.9% of the variance, respectively. PC, principal component; PCA, principal component analysis.

PCA was applied to SG-MSC-corrected cubes for exploratory visualization. PC1–PC3 were used to display dominant spatial variation and to derive LBP and GLCM descriptors; they were not used as the input channels of the deep learning spatial branch. PC1 mainly reflected overall reflectance and tissue-structure variation, while PC2 and PC3 added local edge and gray-level information (Figure 6).

LBP and GLCM features were extracted from the first three PC images to provide interpretable summaries of local texture and gray-level co-occurrence. The analyses were descriptive and were not used to replace the full-band input used by the proposed network.

**Figure 7:**
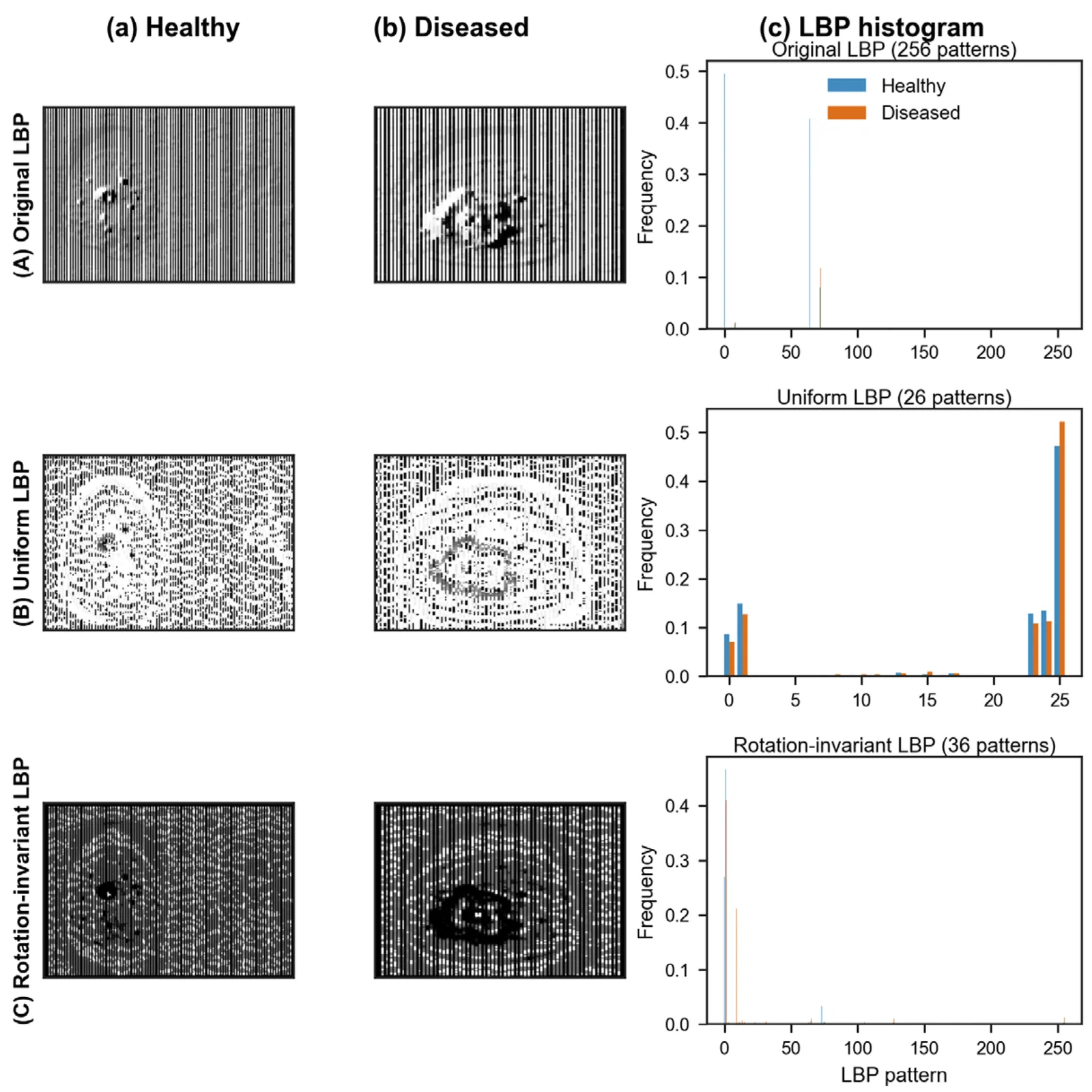
LBP feature maps and histograms for representative healthy and early-infected records. (**A**) Original LBP; (**B**) uniform LBP; (**C**) rotation-invariant LBP; (**a**) healthy; (**b**) early infected; (**c**) pattern frequency histogram. LBP, local binary pattern.

Figure 7 compares original, uniform, and rotation-invariant LBP maps and histograms. Early-infected examples show altered local gray-level patterns and edge aggregation, while the rotation-invariant representation reduces sensitivity to sample orientation. These maps illustrate why spatial texture can complement spectral reflectance.

**Figure 8:**
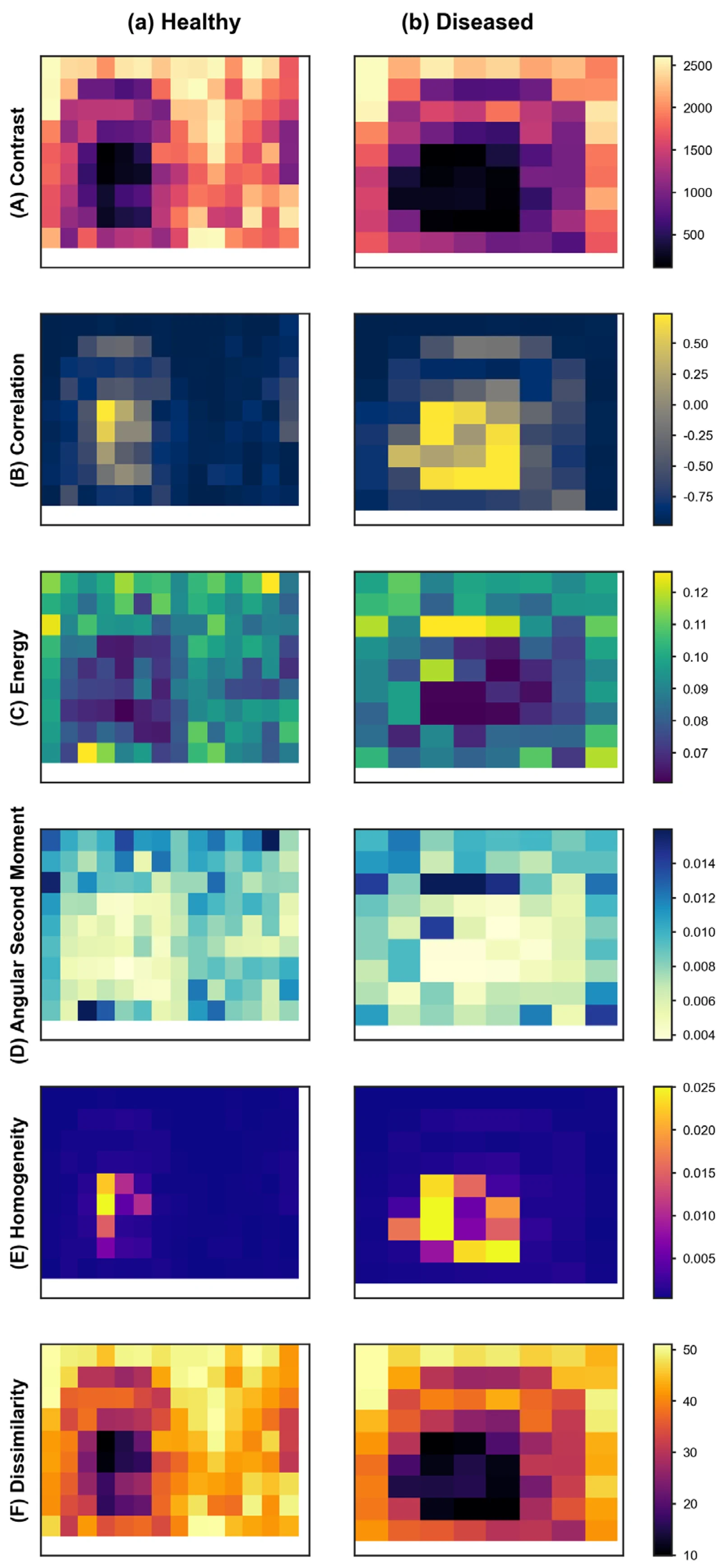
GLCM feature maps for representative tubers: column (**a**), healthy; column (**b**), early infected. Rows show (**A**) contrast, (**B**) correlation, (**C**) energy, (**D**) angular second moment, (**E**) homogeneity, and (**F**) dissimilarity. Color bars indicate the local value of each descriptor. GLCM, gray-level co-occurrence matrix.

Figure 8 and Table 2 show GLCM descriptors calculated from representative PC1 images. The early-infected example has higher correlation, energy, homogeneity, and angular second moment and lower contrast and dissimilarity than the healthy example. These values are descriptive measurements from the displayed images; they are not group-level means, no standard error or standard deviation is implied, and no inferential test is claimed.

The PC images retained visually interpretable spatial differences, and LBP/GLCM summaries showed corresponding changes in local texture and gray-level relationships. These analyses motivate a spatial branch but do not change the full-band input used by the deep learning models.

### 3.3. Single-Feature Benchmarks and Spatial–Spectral Fusion

After SG-MSC preprocessing, conventional spectral benchmarks used fixed competitive adaptive reweighted sampling (CARS) or successive projections algorithm (SPA) band lists with SVM, K-nearest neighbor (KNN), random forest (RF), or backpropagation neural network (BP) classifiers. Table 3 reports the corresponding single-split accuracies. The selected bands were treated as predictive variables; biochemical attribution was limited to broad spectral context rather than a direct metabolite assay.

The conventional models used the following nominal wavelength selections. CARS (39 bands, nm): 1030.3, 1042.9, 1049.2, 1061.9, 1068.2, 1125.0, 1137.6, 1156.5, 1175.5, 1181.8, 1200.7, 1276.4, 1295.3, 1301.6, 1320.6, 1326.9, 1333.2, 1352.1, 1358.4, 1364.7, 1371.0, 1389.9, 1396.2, 1408.8, 1509.6, 1597.7, 1604.0, 1616.6, 1641.8, 1673.3, 1711.0, 1742.4, 1824.2, 1830.5, 1880.7, 1893.3, 1905.9, 1918.4, 1949.8. SPA (16 bands, nm): 1005.0, 1036.6, 1068.2, 1131.3, 1194.4, 1289.0, 1352.1, 1402.5, 1503.3, 1597.7, 1723.6, 1817.9, 1868.2, 1905.9, 1943.6, 1993.8. The wavelength records contain both a nominal 900–1700 nm grid and an extended grid reaching approximately 1994 nm; bands above 1700 nm are therefore reported for reproducibility but are not assigned a biochemical interpretation here. The in-range selections include the water-sensitive region near 1450 nm, while direct attribution to starch, ergosterol, chitin, or another compound would require targeted chemical measurements.

Texture benchmarks used LBP, GLCM, or their fused representation from the PC images. The SVM with fused LBP–GLCM achieved the highest observed accuracy, 91.13% (Table 4). Accuracy was the common endpoint available across texture–model combinations and is reported to two decimal places. These are single-split descriptive benchmarks.

The dual-branch ResNet12-SE model was constructed from the calibrated full-band cube. The spatial branch receives all 224 channels and extracts 256 spatial features; the spectral branch receives the pooled 224-band vector and produces an 8-dimensional sequence feature. Spatial SE, spectral SE, and BiLSTM, respectively, enhance spatial channels, reweight wavelengths, and model dependencies among adjacent bands.

### 3.4. Training Process Analysis of the ResNet12-SE Model

**Figure 9:**
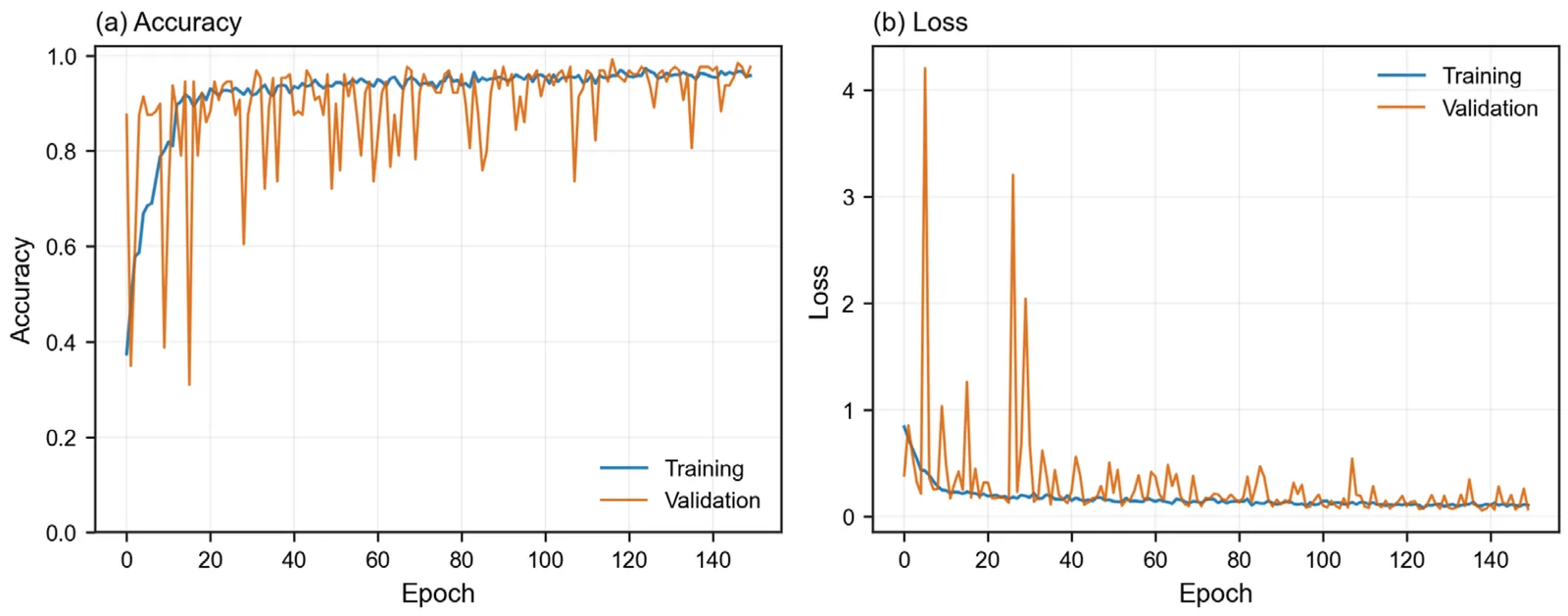
Training and validation curves of the proposed ResNet12-SE model: (**a**) accuracy; (**b**) loss. Blue and orange curves represent the training and validation sets, respectively.

Training and validation accuracy and loss were recorded for one fixed-seed run (Figure 9). Accuracy increased rapidly during the early epochs, while loss decreased. Validation accuracy fluctuated after the initial rise, providing a descriptive measure of performance on the held-out validation subset during training.

The curves approached a plateau after approximately 50 epochs. The implementation trained for 150 epochs and selected checkpoints based on validation accuracy during the final 30 epochs; it did not use a patience-based early stopping rule. The curve therefore documents one training trajectory rather than variability across independent initializations.

### 3.5. Ablation Experiments and Module Contribution Analysis

To examine the contribution of each core module, ablation experiments used the ResNet-12 backbone as the baseline and introduced spatial SE, BiLSTM, and spectral SE separately or jointly (Table 5).

The ResNet-12 backbone reached 90.67% accuracy. Adding spatial SE increased the observed accuracy to 92.24%, a 1.57 percentage point increase. This is consistent with channel recalibration strengthening lesion-related spatial responses, although the result comes from one split.

Adding BiLSTM increased the observed accuracy to 93.79%, 3.12 percentage points above the backbone. The result is compatible with the presence of sequential dependencies across neighboring spectral bands, which a bidirectional recurrent layer can model in both directions.

Adding spectral SE to the ResNet-12 + BiLSTM configuration produced 95.34% accuracy. The module reweighted the 224-band vector and may have reduced the influence of redundant or noisy bands in this run.

Combining spatial SE, BiLSTM, and spectral SE produced the highest observed accuracy, 98.22%, 7.55 percentage points above the backbone. The pattern is consistent with complementary spatial attention, spectral reweighting, and sequence modeling; it should be confirmed with repeated group-wise runs.

The ablation results support the intended modular design under the stated split. They do not establish statistical superiority because repeated seeds, confidence intervals, and paired tests were not calculated.

### 3.6. Comparison with Other Deep Learning Models

**Figure 10:**
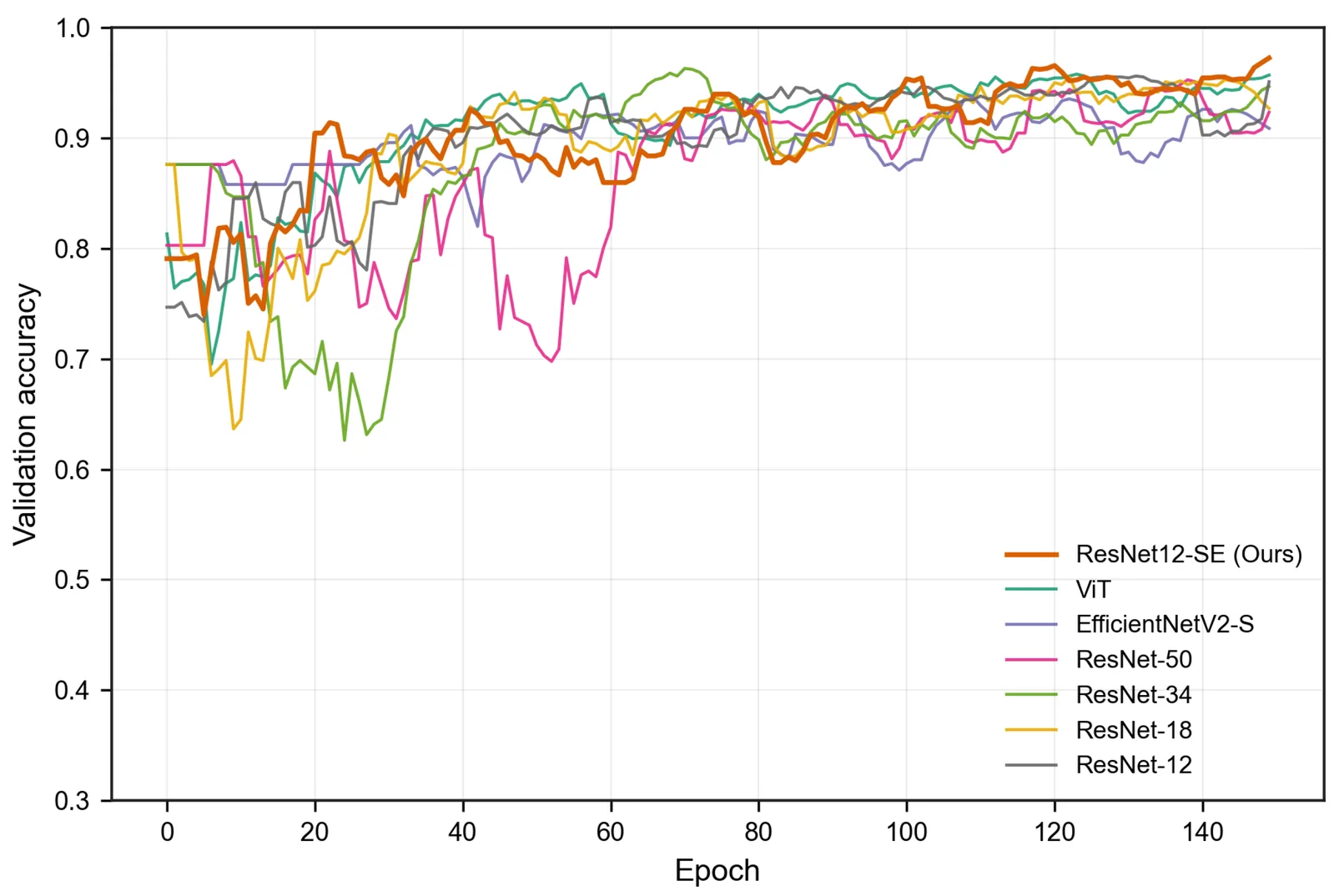
Validation accuracy curves of different deep learning models. Curves show the recorded trajectory for the fixed data split; they are not repeated-run confidence bands.

ResNet-12, ResNet-18, ResNet-34, and ResNet-50 [29] were compared with ResNet12-SE, together with EfficientNetV2-S [30] and Vision Transformer [31]. Every model used the same calibrated 224-band, 224 × 224 input, the same train/validation/test grouping, and the same training schedule; the comparison therefore held input information and partitioning constant. Table 6 reports single-split test accuracy, and Figure 10 shows validation accuracy curves.

ResNet12-SE had the highest observed test accuracy (98.22%), followed by ViT (97.43%). The 0.79 percentage point difference is small relative to the uncertainty expected from a single split, so it is reported as a descriptive benchmark rather than evidence of statistically significant superiority. The comparator models ranged from 92.72% to 94.89% in this comparison, while the ResNet-12 baseline in the ablation analysis was 90.67%.

ResNet-34 performed better than ResNet-18 and ResNet-50 in this run, so deeper networks did not yield a monotonic gain. EfficientNetV2-S and ViT were evaluated with the same hyperspectral input, but neither explicitly models the ordered spectral axis in the way used by the proposed spectral branch.

The proposed model combines full-band spatial encoding with spectral sequence modeling, while the PC images and handcrafted descriptors remain interpretive analyses. This separation makes the input representation explicit and avoids treating three PCA channels as a substitute for the 224-band cube.

## 4. Discussion

Dry rot can begin in subepidermal or internal tissues before clear surface symptoms appear [5]. In this study, the 5% infected volume ratio was used as an operational threshold for a low-volume early infection class. The threshold was selected for the proof-of-concept classification task and should not be interpreted as a universal pathological boundary. Near-infrared hyperspectral imaging captured reflectance and spatial information associated with the labeled states, and the dual-branch model provided a high-accuracy benchmark under the controlled protocol.

The spectral analyses indicate that SG-MSC improved the stability of disease-related reflectance differences, while the SPA-KNN benchmark reached 95.68% accuracy in the reported single split. PC image, LBP, and GLCM analyses showed complementary changes in local texture and gray-level structure, with fused LBP–GLCM features reaching 91.13% in the conventional benchmark. These results support the interpretation that early infection affects both biochemical or water-related reflectance and spatial tissue organization.

ResNet12-SE reached 98.22% test accuracy, the highest observed value among the compared models in this split. Its design combines a 256-dimensional spatial vector with an 8-dimensional spectral sequence vector, and the ablation pattern is consistent with contributions from spatial SE, spectral SE, and BiLSTM. The margin over ViT was 0.79 percentage points; repeated group-wise resampling and paired comparisons are needed to determine whether that difference is reproducible.

Previous spatial–spectral studies have mainly addressed leaves, canopies, or diseases with visible symptoms [24,25,26,32,33,34,35]. The present work focuses on latent postharvest tuber dry rot and uses internal lesion measurements for label confirmation. This focus extends the application context, but it does not establish transferability to naturally infected material or other disease stages.

The current analysis did not stratify prediction errors by continuous lesion volume ratio, so it cannot determine whether misclassifications cluster near the 5% operational cutoff. Boundary cases, weak internal lesions, moisture variation, and puncture-related effects are plausible sources of error. Healthy controls were defined by lesion absence after the stated workflow, and a sterile mock-inoculated mechanical wound group was not separately documented; future experiments should add that control and retain lesion measurements and prediction probabilities for every tuber. Validation was conducted with one cultivar, one purchase batch, one geographic source, one *F. sulphureum* inoculation setting, one laboratory imaging system, and controlled incubation. Robustness across cultivars, naturally infected tubers, pathogen isolates, storage temperatures and durations, geographic sources, calibration states, and acquisition devices remains to be tested with independent external data and repeated group-wise evaluation.

## 5. Conclusions

This study addressed four objectives. (1) Spectral preprocessing and PC image analyses identified distinct reflectance and spatial texture responses associated with early dry rot; SG-MSC was the best-performing preprocessing choice in the single benchmark, and LBP/GLCM provided interpretable spatial descriptors. (2) A dual-branch ResNet12-SE model was constructed using the calibrated 224-band, 224 × 224 cube, with separate spatial and spectral encoding before 264-dimensional feature fusion. (3) Ablation results showed observed gains when spatial SE, spectral SE, and BiLSTM were combined, with 98.22% accuracy in the fixed split. (4) The proposed model had the highest observed accuracy in the comparison with ResNet variants, EfficientNetV2-S, and ViT, although the 0.79 percentage point margin over ViT is descriptive and requires repeated validation. The study supports hyperspectral screening under controlled laboratory conditions and motivates future work with mock wound controls, continuous lesion volume distributions, alternative threshold sensitivity analyses, repeated group-wise testing, multiple cultivars, naturally infected tubers, varied storage conditions, and independent acquisition systems.

## Figures and Tables

**Table 1:** Single-split full-spectrum SVM accuracy under different preprocessing methods. SG, Savitzky–Golay smoothing; MSC, multiplicative scatter correction; SNV, standard normal variate; 1D, first derivative; 2D, second derivative.

Preprocessing Method	SG	2D	1D	SNV	MSC	SG-2D	SG-1D	SG-SNV	SG-MSC
**Accuracy (%)**	93	92	88	58	91	90	94	94	97

**Table 2:** Representative global GLCM descriptors calculated from the displayed PC1 images. The values are descriptive image-level measurements and not group-level estimates. GLCM, gray-level co-occurrence matrix; ASM, angular second moment.

Representative PC1 Image	Contrast	Correlation	Energy	Homogeneity	Dissimilarity	Angular Second Moment
Healthy	73.5762	0.0938	0.1384	0.0142	8.5373	0.0191
Early infected	59.6150	0.4225	0.1473	0.0182	7.6687	0.0216

**Table 3:** Single-split accuracy of conventional models using selected spectral features. CARS, competitive adaptive reweighted sampling; SPA, successive projections algorithm; SVM, support vector machine; KNN, K-nearest neighbor; RF, random forest; BP, backpropagation neural network.

Feature–Classifier	CARS-RF	SPA-SVM	CARS-SVM	SPA-KNN
**Accuracy (%)**	89.45	93.95	94.17	95.68

**Table 4:** Single-split accuracy benchmarks for models using LBP, GLCM, and fused LBP–GLCM texture features. Values are percentages rounded to two decimal places. LBP, local binary pattern; GLCM, gray-level co-occurrence matrix.

Classifier	Feature Set	Accuracy (%)
SVM	LBP	87.71
	GLCM	83.26
KNN	LBP	74.92
	GLCM	82.82
RF	LBP	86.15
	GLCM	84.01
BP	LBP	77.09
	GLCM	70.04
SVM	LBP–GLCM	91.13

All values in Table 4 are single-split accuracy percentages rounded to two decimal places; class-wise metrics were not available for every texture–model combination. Spectral and image features describe complementary aspects of early infection: spectra reflect moisture, tissue composition, and internal scattering, whereas texture descriptors reflect spatial distribution and gray-level structure. These complementary signals motivate separate spectral and spatial encoders followed by feature fusion.

**Table 5:** Single-run ablation accuracies for the ResNet12-SE model under the fixed data split. A check mark (✓) indicates that a module was included, and a cross (×) indicates that it was omitted. SE, squeeze-and-excitation; LSTM, long short-term memory.

ResNet-12	Spatial SE	Bidirectional LSTM	Spectral SE	Single-Run Accuracy (%)
✓	×	×	×	90.67
✓	✓	×	×	92.24
✓	×	✓	×	93.79
✓	✓	✓	×	94.57
✓	×	✓	✓	95.34
✓	✓	✓	✓	98.22

**Table 6:** Single-split test accuracy of deep learning models using the common calibrated 224-band input.

Model	Single-Split Test Accuracy (%)	Model	Single-Split Test Accuracy (%)
ResNet-18	92.72	ResNet-50	93.79
ResNet-34	94.89	EfficientNetV2-S	94.73
ResNet12-SE	98.22	ViT	97.43

## Data Availability

The data presented in this study are available on request from the corresponding authors due to restrictions associated with an ongoing follow-up study covering additional cultivars, naturally infected tubers, and multiple acquisition sites, as further analyses of the same dataset are planned for subsequent publication.

## Abbreviations

The following abbreviations are used in this manuscript:

ASM	Angular second moment
AUC	Area under the receiver operating characteristic curve
BiLSTM	Bidirectional long short-term memory
BN	Batch normalization
BP	Backpropagation neural network
CARS	Competitive adaptive reweighted sampling
CNN	Convolutional neural network
FC	Fully connected
FN	False negative
FP	False positive
GAP	Global average pooling
GLCM	Gray-level co-occurrence matrix
HSI	Hyperspectral imaging
KNN	K-nearest neighbor
LBP	Local binary pattern
LSTM	Long short-term memory
MSC	Multiplicative scatter correction
PC	Principal component
PCA	Principal component analysis
PDA	Potato dextrose agar
ReLU	Rectified linear unit
RF	Random forest
ROC	Receiver operating characteristic
ROI	Region of interest
SE	Squeeze-and-excitation
SG	Savitzky–Golay smoothing
SGD	Stochastic gradient descent
SNV	Standard normal variate
SPA	Successive projections algorithm
SVM	Support vector machine
TN	True negative
TP	True positive
UAV	Unmanned aerial vehicle
ViT	Vision Transformer

## References

[1] Slininger P.J., Schisler D.A., Olsen N.L., Shea-Andersh M.A., Woodell L.K., Hendricks R.L., Miller J.S., Dien B.S. (2025). Control of *Fusarium* dry rot on postharvest Russet Burbank and Clearwater Russet potatoes by *Pseudomonas* biocontrol agents used alone and in combination with a chemical fungicide. Am. J. Potato Res..

[2] Xue H.L., Liu Q.L., Yang Z.M. (2023). Pathogenicity, mycotoxin production, and control of potato dry rot caused by *Fusarium* spp.: A Review. J. Fungi.

[3] Fang G., Yang S., Ruan B., Ye G., He M., Su W., Zhou Y., Wang J., Yang S. (2024). Research progress on physiological, biochemical, and molecular mechanisms of potato in response to drought and high temperature. Horticulturae.

[4] Tan C.R., Tan H.P., He X.Q., Zhao K.W., Zhang W.N., Chen D.G. (2025). Identification of the potato *CR4* gene family and expression pattern analysis in response to dry rot signals. Seed.

[5] Xue H., Bi Y., Wei J., Tang Y., Zhao Y., Wang Y. (2013). New method for the simultaneous analysis of types A and B trichothecenes by ultrahigh-performance liquid chromatography coupled with tandem mass spectrometry in potato tubers inoculated with *Fusarium sulphureum*. J. Agric. Food Chem..

[6] Zhang F., Zhang F.Y., Cui X.H., Wang X.Y., Cao W.H., Zhang Y.K., Fu S.L. (2024). Identification of ginkgo fruit species by hyperspectral image combined with PSO-SVM. Spectrosc. Spectr. Anal..

[7] Mahum R., Munir H., Mughal Z.U.N., Awais M., Sher Khan F., Saqlain M., Mahamad S., Tlili I. (2023). A novel framework for potato leaf disease detection using an efficient deep learning model. Hum. Ecol. Risk Assess..

[8] Shaheed K., Qureshi I., Abbas F., Jabbar S., Abbas Q., Ahmad H., Sajid M.Z. (2023). EfficientRMT-Net—An efficient ResNet-50 and vision transformers approach for classifying potato plant leaf diseases. Sensors.

[9] Li X.T., Zhang F., Feng J. (2024). Convolutional neural network combined with improved spectral processing method for potato disease detection. Spectrosc. Spectr. Anal..

[10] Nguyen C., Sagan V., Maimaitiyiming M., Maimaitijiang M., Bhadra S., Kwasniewski M.T. (2021). Early detection of plant viral disease using hyperspectral imaging and deep learning. Sensors.

[11] Lay L., Lee H.S., Tayade R., Ghimire A., Chung Y.S., Yoon Y., Kim Y. (2023). Evaluation of soybean wildfire prediction via hyperspectral imaging. Plants.

[12] Lu Z., Huang S., Zhang X., Shi Y., Yang W., Zhu L., Huang C. (2023). Intelligent identification on cotton *Verticillium* wilt based on spectral and image feature fusion. Plant Methods.

[13] Bai D.Y., Shi Q.H., Wang J.Q., Sun F.G., Li H.W., Lan P. (2024). Early identification of melon powdery mildew based on hyperspectral feature extraction. J. Chin. Agric. Mech..

[14] Zhang B., Ou Y., Yu S., Liu Y., Liu Y., Qiu W. (2023). Gray mold and anthracnose disease detection on strawberry leaves using hyperspectral imaging. Plant Methods.

[15] Jayswal H., Desai H., Vakani H., Mistry M., Dubey N. (2025). Plant diseases classification with Spectral Signature Taxonomy & Analysis Software (SSTAS). Softw. Impacts.

[16] Hong D., Li C., Yokoya N., Zhang B., Jia X., Plaza A., Gamba P., Benediktsson J.A., Chanussot J. (2026). Hyperspectral imaging. Nat. Rev. Methods Primers.

[17] Joshi V., Snigdha N.S., Aggarwal N. (2026). Early-stage crop disease detection using deep learning. Artificial Intelligence and Sustainable Innovation.

[18] Chavan P., Chavan P.P., Chavan A. (2025). Hybrid architecture for crop detection and leaf disease detection with improved U-Net segmentation model and image processing. Crop Prot..

[19] Ahmad N., Qadri S., Akhtar N. (2024). A multimodal fusion framework to diagnose cotton leaf curl virus using machine vision techniques. Cogent Food Agric..

[20] Sun H., Fu R., Wang X., Wu Y., Al-Absi M.A., Cheng Z., Chen Q., Sun Y. (2025). Efficient deep learning-based tomato leaf disease detection through global and local feature fusion. BMC Plant Biol..

[21] Bala M., Bansal K.S. (2024). Investigating a spectrum of machine learning methods for leaf disease detection in pepper, potato, and tomato. ECS J. Solid State Sci. Technol..

[22] Kuswidiyanto L.W., Wang P., Noh H.H., Jung H.-Y., Jung D.-H., Han X. (2023). Airborne hyperspectral imaging for early diagnosis of kimchi cabbage downy mildew using 3D-ResNet and leaf segmentation. Comput. Electron. Agric..

[23] Yang M. (2022). Physiological disorder diagnosis of plant leaves based on full-spectrum hyperspectral images with convolutional neural network. Horticulturae.

[24] Zhong X., Li H., Cai Y., Deng Y., Xu H., Tian J., Liu S., Hou M., Weng H., Wang L. (2025). Early Detection of Tomato Gray Mold Based on Multispectral Imaging and Machine Learning. Horticulturae.

[25] Cheng X., Huang W., Guo A., Cai Z., Dong Y., Hu B., Chen G., Su H., Li L. (2026). Monitoring of rubber tree powdery mildew by combining spatial-spectral features and plant traits quantified from UAV hyperspectral imagery. Comput. Electron. Agric..

[26] Tan F., Cang H., Gao X., Wu N., Di R., Zhang Y., Gao P., Lv X., Zhang C. (2025). Early detection of cotton *Verticillium* wilt based on generative adversarial networks and hyperspectral imaging technology. Ind. Crops Prod..

[27] Syahrial S., Melinda M., Junidar J., Razali S., Zulhelmi Z. (2024). Application of the Savitzky–Golay filter in multi-spectral signal processing. Sriwij. Electr. Comput. Eng. J..

[28] Maliza N.O., Sulaiman M.I., Yunita D., Munawar A.A., Andini R., Safrida (2026). Multiplicative scatter correction improves near-infrared spectroscopy-based PLS models for acetic acid quantification in jamblang vinegar. BIO Web Conf..

[29] He K., Zhang X., Ren S., Sun J. Deep residual learning for image recognition. Proceedings of the IEEE Conference on Computer Vision and Pattern Recognition.

[30] Tan M., Le Q. (2021). EfficientNetV2: Smaller models and faster training. Proceedings of the International Conference on Machine Learning, Virtual Event, 18–24 July 2021.

[31] Dosovitskiy A., Beyer L., Kolesnikov A., Weissenborn D., Zhai X., Unterthiner T., Dehghani M., Minderer M., Heigold G., Gelly S. (2020). An image is worth 16 × 16 words: Transformers for image recognition at scale. arXiv.

[32] Jung D.H., Kim J.D., Kim H.Y., Lee T.S., Kim H.S., Park S.H. (2022). A hyperspectral data 3D convolutional neural network classification model for diagnosis of gray mold disease in strawberry leaves. Front. Plant Sci..

[33] Yan T., Xu W., Lin J., Duan L., Gao P., Zhang C., Lv X. (2021). Combining multi-dimensional convolutional neural network (CNN) with visualization method for detection of *Aphis gossypii* Glover infection in cotton leaves using hyperspectral imaging. Front. Plant Sci..

[34] Ashwini C., Sellam V. (2024). An optimal model for identification and classification of corn leaf disease using hybrid 3D-CNN and LSTM. Biomed. Signal Process. Control.

[35] Gao W., Xiao Z., Bao T. (2023). Detection and identification of potato-typical diseases based on multidimensional fusion Atrous-CNN and hyperspectral data. Appl. Sci..

